# Addiction to ultra-processed foods as a mediator between psychological stress and emotional eating during the COVID-19 pandemic

**DOI:** 10.1186/s41155-024-00322-1

**Published:** 2024-09-18

**Authors:** Jasmin B. Stariolo, Thayane C. Lemos, Neha Khandpur, Mirtes G. Pereira, Leticia de Oliveira, Izabela Mocaiber, Taiane C. Ramos, Isabel A. David

**Affiliations:** 1 Department of Physiology and Pharmacology, Biomedical Institute, Universidade Federal Fluminense, Niterói, Brazil; 2 Department of Nutrition, Center for Epidemiological Research in Nutrition and Health, Universidade de São Paulo, São Paulo, Brazil; 3 Department of Nutrition, Harvard T.H. Chan School of Public Health, Harvard University, Boston, MA USA; 4 Laboratory of Cognitive Psychophysiology, Department of Natural Sciences, Institute of Humanities and Health, Universidade Federal Fluminense, Rio das Ostras, Brazil; 5 Department of Computer Science, Computer Institute, Universidade Federal Fluminense, Niterói, Brazil

**Keywords:** Food addiction, Emotional eating, Psychological stress, Mediation analysis, COVID-19

## Abstract

**Background:**

The coronavirus disease 2019 (COVID-19) pandemic induced psychological distress, which is linked to emotional eating and symptoms of addiction to ultra-processed foods (UPFs).

**Objective:**

This study aimed to investigate whether symptoms of addiction to UPFs mediate the relationship between psychological stress due to the COVID-19 pandemic and emotional eating behaviour.

**Methods:**

A cross-sectional online study was conducted from May to November 2021 among 368 undergraduate Brazilian students. The participants answered demographic questions and completed validated scales, including the Coronavirus Stress Measure, Modified Yale Food Addiction Scale 2.0 and Emotional Eating Questionnaire. Mediation analysis was employed to examine the hypothesised relationships.

**Results:**

The results revealed a significant indirect effect, indicating that symptoms of food addiction mediated the association between perceived stress during the COVID-19 pandemic and emotional eating behaviour. Specifically, 61% of the influence of perceived stress on emotional eating during the pandemic was explained by symptoms of UPF addiction.

**Conclusion:**

These findings suggest that addressing symptoms of UPF addiction could be pivotal in public health strategies aimed at promoting healthy eating habits among distressed undergraduate students in the post-COVID-19 era.

**Supplementary Information:**

The online version contains supplementary material available at 10.1186/s41155-024-00322-1.

## Introduction

### COVID-19 pandemic, psychological stress and emotional eating

At the end of 2019, the coronavirus disease 2019 (COVID-19) pandemic caused by the SARS-CoV-2 virus began worldwide, with impacts on individuals’ physical and mental health (Cénat et al., 2021; Portugal et al., 2022). The COVID-19 pandemic’s health risks and containment measures had a significant impact on mental health, increasing individuals’ psychological stress (Brooks et al., 2020).

Psychological stress was defined by Folkman (2013) as ‘…a particular relationship between the person and the environment that is appraised by the person as taxing or exceeding his or her resources and endangering his or her well-being’ (Folkman, 2013, p. 19). Folkman (2013) posits that an individual’s assessment of a situation influences their stress responses and coping mechanisms. From this situational perspective, coping is described as ‘cognitive and behavioural efforts to master, reduce, or tolerate the internal and/or external demands that are created by the stressful transaction’ (Folkman, 1984, p. 843). While the COVID-19 pandemic affected the entire population, the devastating effects of psychological stress on mental health varied greatly among individuals, which may indicate that resilience and/or coping mechanisms differ among individuals (Bhattacharjee & Ghosh, 2022; Portugal et al., 2022).

Psychological stress may trigger neurobiological adaptations that usually affect food-related motivational brain circuits, with consequences for eating behaviour (Yau & Potenza, 2013). Indeed, Liboredo and colleagues reported that psychological stress during the COVID-19 pandemic was associated with emotional eating and increased food intake in a Brazilian sample (Liboredo et al., 2021). Emotional eating is usually defined as the propensity to consume large quantities of food due to feelings rather than hunger (Frayn & Knäuper, 2018). Emotional eating behaviour may occur because some individuals use food as a way of coping with stress, resulting in overeating and unhealthy diets (Devonport et al., 2019). Thus, the increase in negative emotions and stress levels during the COVID-19 pandemic was expected to lead to emotional eating behaviour (Cheng & Wong, 2021; Madalı et al., 2021). Emotional eating symptoms have also been found to be associated with a greater likelihood of binge eating disorders (Rossi et al., 2020). In fact, stressful events due to the COVID-19 pandemic have been positively associated with binge-eating behaviours (Miniati et al., 2021).

### Food addiction and emotional eating

In addition to psychological stress, Rossi and colleagues reported that food addiction is related to emotional eating (Rossi et al., 2020). Food addiction is considered a substance-based addiction to ultra-processed foods (UPFs), especially since not all foods are potentially addictive (Gearhardt & Schulte, 2021; Monteiro & Cannon, 2023; Schulte et al., 2015). According to the NOVA classification system, UPFs are composed mainly of industrial sources of dietary energy and nutrients plus additives (Monteiro et al., 2016, 2019). There is evidence that UPFs may be capable of triggering addictive behaviour (Gearhardt & Schulte, 2021; Monteiro & Cannon, 2023; Schulte et al., 2015). UPFs are rich in calories, are associated with aggressive marketing and are hyperpalatable, which makes them much more attractive than unprocessed or minimally processed foods (Lemos et al., 2022; PAHO, 2019b).

UPFs undergo several industrial processing stages that promote physical and chemical changes that drive addictive behaviour, such as decreased water content, which also decreases satiety, and excess chemical additives, sugar, salt and caffeine (Gordon et al., 2018; Lustig, 2020). The combination of the physical and chemical characteristics of UPFs can potentially lead to neurobiological and psychological behavioural impairments associated with substance addiction similar to drug addiction. Some of those symptoms include opioid and dopamine overexpression along with withdrawal, tolerance and loss of control (Gearhardt & Schulte, 2021; Gordon et al., 2018).

The psychological and behavioural impairments linked to UPF consumption meet the Diagnostic and Statistical Manual of Mental Disorders (DSM-5) criteria for substance use disorder, even though food addiction is not currently classified as a condition in the DSM-5. Although evidence supporting the concept of addictive-like eating is growing, a crucial point of debate is classifying foods as addictive to have food addiction included in the DSM-5 (Gearhardt et al., 2023). The literature has just recently begun to discuss foods, particularly those that may cause addiction, on the basis of the NOVA classification system (Gearhardt & Schulte, 2021; Gearhardt et al., 2023). As a result, UPFs are increasingly recognised as important food addiction triggers, which could eventually lead to the inclusion of food addiction in the DSM-5.

A growing body of evidence has suggested that food addiction to UPFs may help in understanding the neural and behavioural mechanisms involved in maladaptive coping strategies for emotional distress, including emotional eating (Gearhardt et al., 2011; Pape et al., 2021; Parylak et al., 2011; Rossi et al., 2020; Whiteside et al., 2007). A better understanding of the relationship between addiction to UPFs and emotional eating is important. Although emotional eating has been traditionally defined as overeating in response to negative emotions, the experience of negative emotions does not necessarily increase food intake (Nolan et al., 2010). For example, some individuals may report eating more when they experience distress, whereas others may report eating less under the same condition (Nolan et al., 2010). Symptoms of food addiction are substantially greater in individuals who overeat than in individuals who do not (Ng & Davis, 2013). Thus, symptoms of addiction to UPFs may act as potent risk factors for overeating behaviours and serve as a common trigger for the development of emotional eating and binge eating disorders (Gearhardt et al., 2012). Symptoms of addiction to UPFs could help in understanding how an individual’s appetite can sometimes become excessive in response to negative feelings.

### Food addiction and psychological stress

Stress seems to induce dysregulation of motivational systems in the brain, which increases the risk of food addiction (Wei et al., 2019). For individuals experiencing stress, eating can alter the neuroendocrine balance (e.g. dysregulation of the hypothalamic–pituitary–adrenocortical axis), which can further sensitise the brain’s reward centre and create a positive feedback loop to sustain opioid stimulation from hyperpalatable foods, such as UPFs (Adam & Epel, 2007; Wei et al., 2019). Stress and food addiction behaviour may therefore be a feed-forward process. Thus, it is likely that psychological stress following the COVID-19 pandemic may trigger the urge to consume UPFs. In turn, UPFs may comfort individuals in a stressed state (Orford, 2001), increasing their food consumption in response to stress (i.e. emotional eating) (Macht, 2008).

In fact, previous studies have shown a relationship between food addiction and negative urgency, i.e. a propensity to behave hastily when experiencing negative emotions (Minhas et al., 2021). A negative reinforcement model, in which people eat to temporarily feel better after experiencing a negative emotion, could help explain the association between food addiction and negative urgency (Fischer et al., 2004). Importantly, although the focus here is on negative feelings (because the COVID-19 pandemic was a negative event), food addiction has also been linked to positive urgency (Minhas et al., 2021). In this case, individuals tend to act rashly when experiencing positive emotions and may eat more in response to positive moods. However, it is likely that food addiction symptoms are more strongly linked to relief from negative affective states, such as psychological stress states, than to the pleasant feelings that the consumption of food provides (Meule & Kübler, 2012).

Nearly everyone was impacted by the COVID-19 pandemic, either directly or indirectly, either as a result of COVID-19 infection (or fear of infection) or as a result of the effects of far-reaching measures and their implications for the economy and society. As a result, these multiple stress factors impacted individuals’ food addiction symptoms (da Silva Júnior et al., 2022; Panno et al., 2020; Zielińska et al., 2021). Many studies have shown an association between food addiction and psychological stress (Beyer et al., 2019; Hong et al., 2020; Hussenoeder et al., 2024; Lin et al., 2020; Najem et al., 2020). Hong and colleagues reported that psychological stress and food addiction are not just related: In fact, psychological stress can predict the development of food addiction symptoms (Hong et al., 2020). That is, both psychological stress and food addiction are linked at the level of addictive symptoms.

### Purpose of the present study

The rationale here is that since food addiction interacts with both emotional eating and psychological stress related to the COVID-19 pandemic, these three factors should be accounted for collectively. Previous studies have independently examined the associations between emotional eating or food addiction and psychological stress during the COVID-19 pandemic (Cheng & Wong, 2021; da Silva Júnior et al., 2022; Liboredo et al., 2021; Madalı et al., 2021; Panno et al., 2020; Zielińska et al., 2021). The associations between food addiction and both psychological stress and emotional eating suggest a potential mediating role of food addiction that may help clarify the relationship between stress and emotional eating. However, studies examining the relationships among food addiction, emotional eating and psychological stress during the COVID-19 pandemic in a mediation model have not been conducted. Here, we hoped to close this knowledge gap and gain new insights into how these factors interact.

**Fig. 1:**
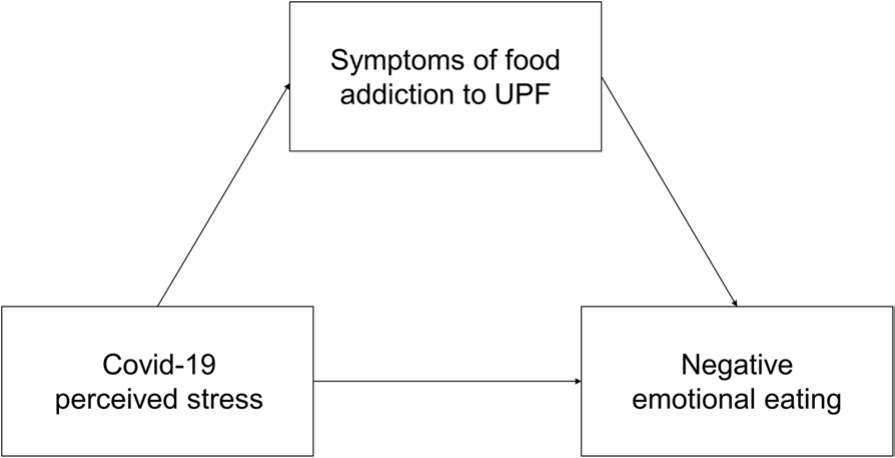
Proposed model: mediating role of symptoms of addiction to ultra-processed foods (UPFs) in the relationship between perceived COVID-19-related stress and negative emotional eating

We investigated whether symptoms of addiction to UPFs may be important factors in mediating emotional eating behaviours, especially when individuals are experiencing negative emotions, such as during the COVID-19 pandemic. We hypothesised that food addiction symptoms could be a significant mediator between a stressful event and negative emotional eating behaviour (see Fig. 1).

Understanding this mediating role of symptoms of addiction to UPFs as a potential underlying mechanism of the relationship between stress during the COVID-19 pandemic and emotional eating may be useful in supporting public health strategies such as reducing the availability and of UPFs in food environments and their consumption. Repeated and intermittent intake of UPFs may trigger symptoms of eating disorders, such as food addiction and emotional eating. In combination with strategies to reduce stress, promoting healthier food environments may prevent emotional eating.

## Materials and methods

### Participants and procedure

A cross-sectional study using a web survey was conducted between May and November 2021. Data were collected via a convenience snowball sampling technique. The study targeted a convenience sample of undergraduate students from a public university in Brazil. During this period, the students were engaged in virtual learning due to pandemic restrictions. Participants were recruited through email, Instagram, WhatsApp and Facebook. Interested individuals received an email containing a link to the survey hosted on Google Forms and were instructed to complete a series of questionnaires.

We focused on a sample of college students because college is an important time in early adulthood in which individuals are more susceptible to body weight gain and the adoption of unhealthy eating habits (Vadeboncoeur et al., 2015). Many college students change their eating behaviour as a coping strategy to address psychological stress (Bennett et al., 2013; Penaforte et al., 2016). Before the COVID-19 pandemic, more than 50% of Brazilian college students presented symptoms of stress (Padovani et al., 2014; Penaforte et al., 2016). During the COVID-19 pandemic, this prevalence increased even more (Lopes & Nihei, 2021) because of social isolation, worries and uncertainties about academic and personal life during this period (Sahu, 2020).

**Fig. 2:**
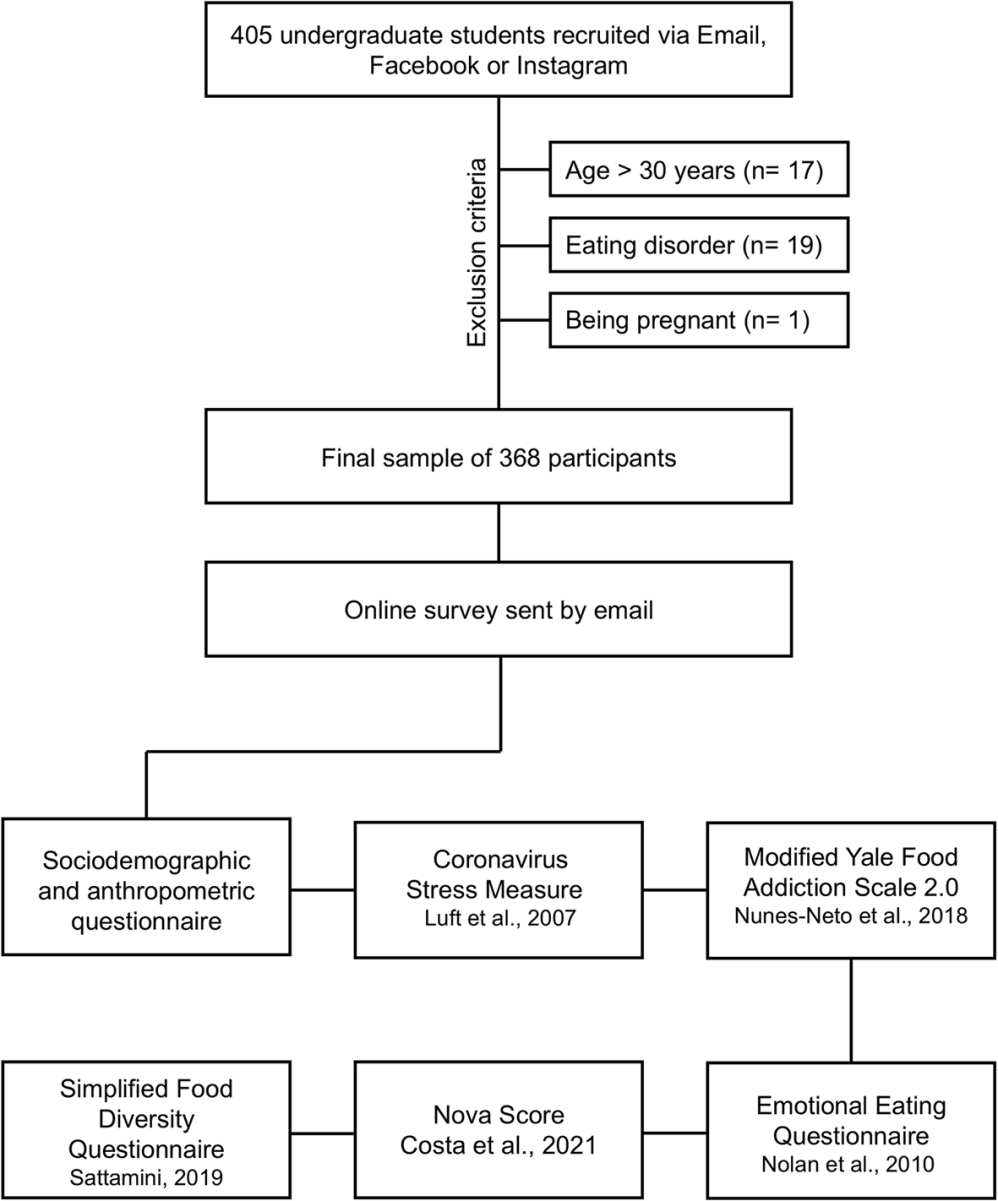
Sample size after applying the exclusion criteria. The order in which the questionnaires were applied is also described in the bottom panel

The eligibility criteria included (1) individuals between 18 and 30 years of age, (2) individuals who were native Portuguese speakers and (3) individuals who had not been diagnosed with eating disorders and were not pregnant. We excluded individuals diagnosed with eating disorders because the prevalence of food addiction among individuals with binge eating, bulimia and anorexia nervosa is greater than that among nonclinical populations (Wiss, 2022). The criteria for exclusion and the applied questionnaires are illustrated in Fig. 2.

To determine the sample size, we aimed for a 95% confidence level with a 5% margin of error, considering a population of approximately 40,000 undergraduate students at the university. This calculation suggested a required sample size of 381 participants (Santos, 2017). Ultimately, 405 students completed the survey. After applying the eligibility criteria, the final sample consisted of 368 participants aged 18 to 30 years (mean age = 21.8 years; standard deviation = 2.9 years).

Most of the participants were women (66.8%) and Caucasian (59.5%). Students with social vulnerability who receive a monthly income of less than approximately 1.5 times the minimum wage in Brazil accounted for 33.3% of the sample. Students from all major knowledge areas participated in the study, with social science (44%) being the most representative, as described in Table 1. The mean BMI calculated from the reported weight and height was 23.1 kg/m^2^ (SD = 4.6; BMIMin = 14.8 kg/m^2^; BMIMax = 43.1 kg/m^2^; see Table 1).

**Table 1:** Sociodemographic characteristics of the college students (*n* = 368)

	*N* (%)
**Gender**	
Women	246 (66.8)
Men	116 (31.5)
Nonbinary or fluid	6 (1.6)
**BMI (kg/m**^**2**^**)**	
Underweight (< 18.5)	42 (11.4)
Eutrophic (18.5–24.9)	228 (61.9)
Overweight (25–29.9)	64 (17.4)
Obese (≥ 30)	34 (7.8)
**Social vulnerability**^**a**^	
Yes	122 (33.1)
No	246 (66.9)
**Area of knowledge**	
Social science	161 (44)
Health science	99 (27)
Hard science	50 (14)
Engineering	32 (9)
Biological science	26 (7)
**Race/ethnicity**	
White	219 (59.5)
Black	142 (38.5)
Asian	3 (0.8)
Indigenous	0 (0.0)
Not informed	4 (1.0)

^a^Social vulnerability concerns people with less than 1.5 times the minimal household income. The body mass index (BMI) cut-off was based on the WHO (2000)

### Measures

#### Questionnaires and psychometric scales

The Coronavirus Stress Measure (CSM) (Arslan et al., 2021) is an adaptation of the Perceived Stress Scale (Cohen et al., 1983) that was adapted and validated in Portuguese by Luft et al. (2007). Developed to specifically measure how the COVID-19 pandemic affected individuals’ perceived psychological stress, this scale contains eight items referring to how often a person felt a certain way, specifically due to the COVID-19 pandemic (e.g. ‘How often were you upset because of the COVID-19 pandemic?’). Thus, the scale was designed to assess perceptions of COVID-19-related psychological stress. The CSM is scored on a 5-point scale ranging from 0 (never) to 4 (always); thus, the total score varies from 0 to 32 points. The higher the total score is, the greater the perceived psychological stress during the COVID-19 pandemic. The internal consistency measured by Cronbach’s alpha in the present study was 0.86.

The Modified Yale Food Addiction Scale 2.0 (mYFAS 2.0) (Schulte & Gearhardt, 2017) has thirteen questions regarding eating behaviour in the past 12 months (the questions can be viewed in supplemental material). The scale was adapted and validated in Portuguese by Nunes-Neto et al. (2018). The mYFAS 2.0 aims to assess addictive-like eating behaviour on the basis of the current addiction diagnostic criterion described in the DSM-5. It provides two final scores: (1) a continuous symptom count that reflects the number of addiction-like criteria endorsed and (2) a diagnostic criterion with four classifications: ‘no food addiction’, ‘mild food addiction’, ‘moderate food addiction’ and ‘severe food addiction’ (Gearhardt et al., 2016). Only the symptom count score was used in the mediation analysis. Half of individuals may present at least one symptom of food addiction (Koehler et al., 2021). From a psychiatric perspective, it is important to focus on the diagnosis to develop better treatment procedures. However, from a public health point of view, focusing on the number of continuous symptoms of food addiction is important to prevent food addiction behaviours in the overall population, for example by promoting public health measures to reduce UPF intake. The internal consistency measured by Cronbach’s alpha in the present study was 0.88.

The original Emotional Eating Questionnaire (EMAQ) was developed by Geliebter and Aversa (2003) and validated by Nolan et al. (2010). The instrument was also adapted and validated in Portuguese by Sabry et al. (2020). The EMAQ aims to assess how certain emotional states and situations affect individuals’ eating behaviour. The Brazilian Portuguese version (Sabry et al., 2020) includes 22 original items: 14 items related to emotions (9 negative and 5 positive emotions such as sadness, anger, anxiety, happiness and relaxation) and 8 items related to situations (5 negative and 3 positive situations, such as after ending a relationship or falling in love). Participants are asked to mark how much they tend to eat for each emotion or situation on a Likert-type scale ranging from 1 (‘much less’) to 9 (‘much more’).

Unlike other emotional eating scales in which emotional eating is viewed as an overeating response to negative emotions, the EMAQ considers the possibility that individuals may eat more when they are happy or eat less when they are sad. Thus, the EMAQ not only focuses on negative emotions but also accounts for positive emotions. This aspect of the EMAQ is important since food addiction was previously correlated with positive urgency, in which positive moods may also lead to overeating behaviour (Minhas et al., 2021). In addition, the EMAQ considers that certain emotional states may cause less food intake than usual rather than more food intake. We obtained two final scores from the EMAQ: the arithmetic mean of (1) negative emotion responses and that of (2) positive emotion responses. The internal consistency for the present study, as assessed by Cronbach’s alpha, was high for the whole EMAQ scale (0.88), as were the internal consistency for the negative emotions/situations (0.91) and positive emotions/situations (0.90).

The Nova score (Costa et al., 2021) is based on the Nova classification (Monteiro et al., 2016) and aims to evaluate the consumption of UPFs. In total, the scale contains 23 subgroups of UPFs, which are divided into three main categories: beverages (6 subgroups), products that replace or go with meals (10 subgroups) and snacks (7 subgroups). Participants are asked to select the items that they consumed the day before. The final score corresponds to the sum of all selected items in the three categories.

The last questionnaire used was the Simplified Food Diversity Questionnaire (Sattamini, 2019), which has 10 unprocessed food categories from group 1 of the Nova classification system (Monteiro et al., 2016). Participants select the categories that contain a food item consumed the day before; thus, the final score ranges from 0 to 10 points. This questionnaire was added to the study, in addition to the Nova score (mentioned above), to confirm that food addiction is related only to the consumption of UPFs and is not related to the consumption of unprocessed or minimally processed foods, as previously described (Schulte et al., 2015).

### Statistical analysis

We performed descriptive statistical analyses of the data, including the means and standard deviations of the participants’ scores obtained from the questionnaires. The data presented a nonnormal distribution, as assessed by the D’Agostino–Pearson test (*p* < 0.05) for all the variables.

There were no missing data between and within participants. Answers from all the items of all the measures could be assessed and analysed. Because the psychometric instruments used were completed online, i.e. a different form of application in relation to the validated versions of the scales, we conducted a confirmatory factor analysis (CFA) to examine the construct validity of the scales for the present sample. The results of these analyses are presented in the supplemental material.

To confirm that our data were in line with the well-established association in the literature (Gearhardt & Schulte, 2021; Monteiro & Cannon, 2023; Schulte et al., 2015), we tested whether food addiction symptoms are related to the consumption of UPFs and not to the consumption of unprocessed or minimally processed foods. Since these data presented a nonnormal distribution, we calculated the Spearman correlation between these variables separately. The significance level was Bonferroni adjusted to an alpha < 0.025.

**Fig. 3:**
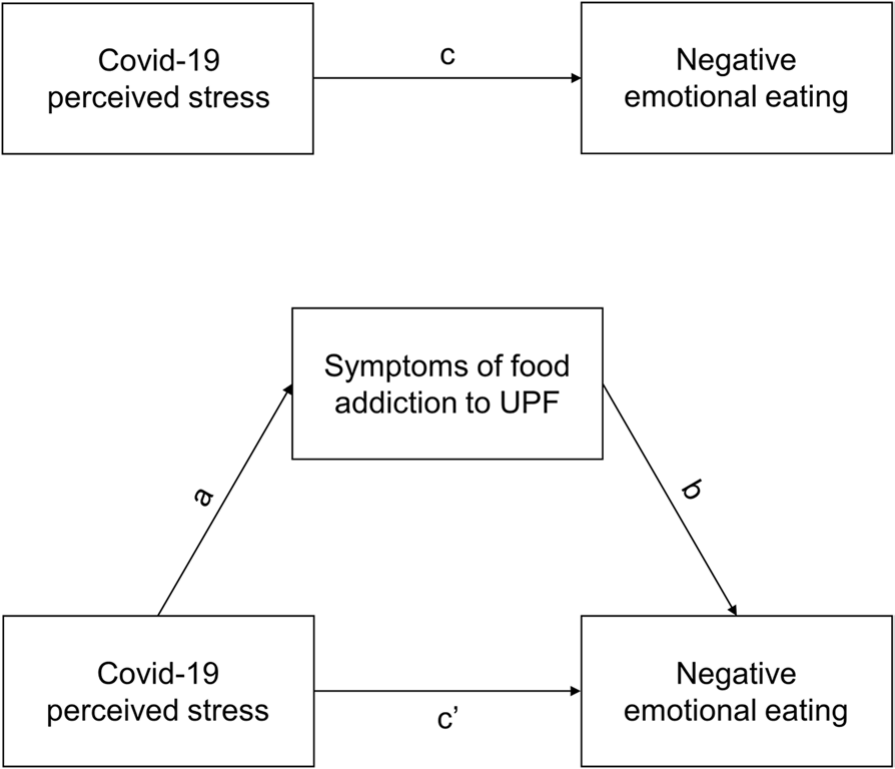
Path diagram for the proposed mediation model in which symptoms of addiction to ultra-processed foods (UPFs) mediate the effect of perceived COVID-19-related stress (independent variable) on negative emotional eating (dependent variable). The mediation analysis consists of three linear regressions that estimate the total effect (c path), indirect effect (a*b path) and direct effect (c’ path)

Figure 3 shows the mediation analysis model, in which the independent variable is the final score of the *CSM*, named ‘COVID-19 perceived stress’; the dependent variable is the final score of the EMAQ, named ‘negative emotional eating’; and the mediator is the final continuous score of the mYFAS 2.0, named ‘symptoms of addiction to UPFs’ (MacKinnon & Fairchild, 2009). Even though it was possible to test more complex models with multiple mediators, the focus here was to provide a better understanding of food addiction symptoms as a behavioural risk factor that mediates the relationship between perceived stress due to the COVID-19 pandemic and emotional eating symptoms.

To establish a mediating effect, three conditions must be satisfied: (i) a significant path a, b and c; (ii) a significant indirect effect (a*b); and (iii) a direct effect (c’) smaller than the total effect (c) (i.e. |c| >|c’|) (MacKinnon & Fairchild, 2009). If the three conditions were satisfied, it would be possible to assume that there is a mediating effect of symptoms of addiction to UPFs on the relationship between perceived COVID-19-related stress and negative emotional eating.

To investigate the significance of the indirect effect, we performed a bootstrap procedure. Indirect effects were computed for each of the 1000 bootstrap samples. The adopted coefficient significance level was an alpha = 0.05, and the 95% confidence interval was computed for the effects by determining the indirect effect at the 2.5th and 97.5th percentiles. All analyses were performed with R software version 2021.09.2 (R Development Core Team, 2022) via the ‘mediation’ package. The same analysis was conducted via Hayes’ PROCESS Model 4, and the results remained the same (see the Supplemental material).

### Confounders

We performed Spearman correlations between EMAQ data (dependent variable) and household income, race, sex and BMI to identify possible confounders or variables that could explain a mediating role in addition to symptoms of food addiction. Only EMAQ items related to negative emotions were analysed since the period of interest in this study was during the COVID-19 pandemic. The significance level was Bonferroni adjusted to an alpha < 0.015.

BMI presented a significant correlation with the EMAQ score in our study (rho = 0.298; *p* < 0.001). Hence, before performing the mediation analysis, we regressed out the effect of the EMAQ score from the three variables used in the analysis (namely, perceived COVID-19-related stress, symptoms of addiction to UPFs and negative emotional eating) to exclude any potential confounding effects. BMI was calculated from the reported height and weight.

Apart from BMI (rho = 0.298; *p* < 0.001), there was no correlation between the EMAQ score and household income (rho =  − 0.028; *p* = 0.59), race (rho = 0.04; *p* = 0.45) or sex (rho = 0.006; *p* = 0.91). Therefore, to account for confounders, only BMI was regressed out of the mediation analysis.

## Results

Before the mediation analysis, we checked the relationships between the variables of interest. Table 2 shows that even after Bonferroni correction, all three variables of interest for the mediation analysis (perceived COVID-19-related stress, symptoms of addiction to UPFs and negative emotional eating) were positively correlated. When experiencing negative emotions, individuals who scored higher on the food addiction (Mann‒Whitney *U* (10,227),* p* < 0.001) and perceived COVID-19-related stress (*U* (12,913),* p* = 0.016) scales tended to overeat more (i.e. score greater than five on the negative emotional eating scale) than those who typically ate less (i.e. score lower than five on the negative emotional eating scale).

**Table 2:** Mean (M), standard deviation (SD), minimum (Min.), maximum (Max.) and Spearman correlation matrix (rho)

Variables	M	SD	Min	Max	1	2	3	4
COVID-19 perceived stress (1)	14.8	4.3	0.0	20.0	–			
Symptoms of food addiction to UPF (2)	1.3	1.8	0.0	10.0	0.30^*^	–		
Negative emotional eating (3)	4.8	1.8	1.0	9.0	0.14^*^	0.35^*^	–	
Positive emotional eating (4)	4.8	1.7	1.0	8.8	− 0.003	− 0.07	− 0.20^*^	–

^*^p < 0.008

Importantly, only the responses to the EMAQ instrument related to negative emotions (negative emotional eating variable) were positively correlated with perceived COVID-19-related stress (rho = 0.14; *p* < 0.008) and symptoms of addiction to UPFs (rho = 0.35; *p* < 0.008). The responses related to positive emotions (positive emotional eating variable) did not correlate with symptoms of addiction to UPFs (rho =  − 0.07; *p* = 0.18) nor with COVID-19-related stress (rho =  − 0.003; *p* = 0.96). This result reinforced the use of negative emotional eating instead of positive emotional eating during the mediation analysis.

**Fig. 4:**
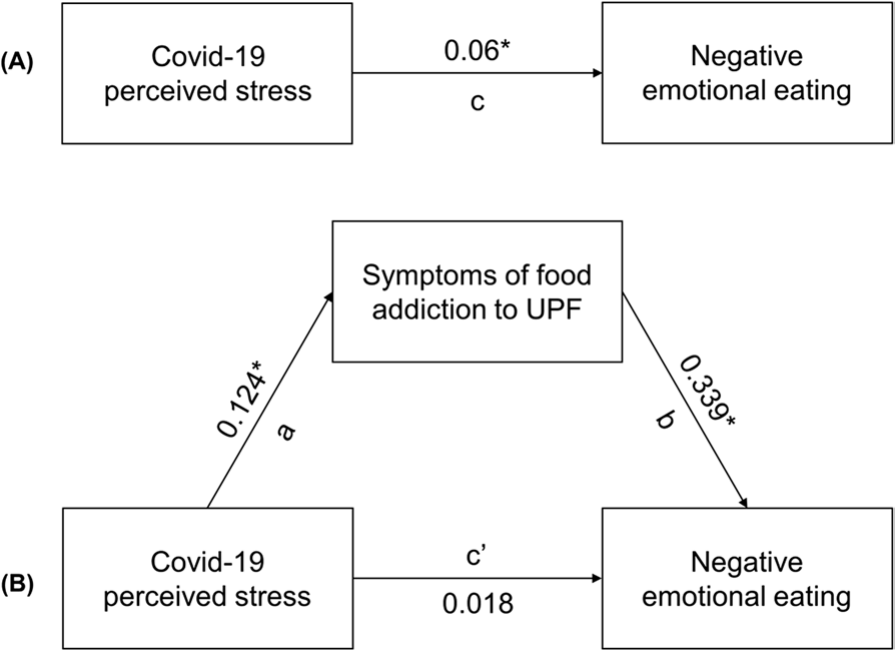
Path diagram of the mediation model showing the three paths for estimating the total effect. The beta scores obtained are shown for each path. **A** The total effect (path c). **B** The effect of COVID-19-related stress on food addiction symptoms (path a); the effect of food addiction symptoms on negative emotional eating, adjusted for COVID-19-related stress (path b); and the direct effect of the independent variable (COVID-19-related stress) on the dependent variable (negative emotional eating), adjusted by the mediator (path c’). *p < 0.008

The effect of perceived stress due to the COVID-19 pandemic on negative emotional eating was mediated by symptoms of addiction to UPFs, even after correction for possible confounders. Figure 4 shows all the linear regression coefficients obtained for each path. Scatter plots with fitted linear regression lines are provided in Supplemental material.

The influence of COVID-19-related stress on negative emotional eating was significant (path c = 0.06; *p* = 0.01; prerequisite 1) when the mediating effect was not considered (Fig. 4A). On the other hand, the relationship between COVID-19-related stress and negative emotional eating was no longer significant after the mediating effect (symptoms of addiction to UPFs) was removed (path c’ = 0.02; *p* = 0.3). Finally, the linear regression coefficient between perceived COVID-19-related stress and symptoms of addiction to UPFs (a) and between symptoms of addiction to UPFs and negative emotional eating (b) was significant [(a) = 0.124; *p* < 0.001; (b) = 0.339; *p* < 0.001] (prerequisite 2, Fig. 4B).

The indirect effect was calculated by multiplying ‘a’* ‘b’, i.e. (0.124)*(0.339) = 0.042, and by performing the bootstrapping procedure, the mean was calculated as 0.042. The 95% confidence interval was computed by determining the indirect effects at the 2.5th and 97.5th percentiles, obtaining results varying from 0.02 to 0.05. Thus, the indirect effect was statistically significant (*p* < 0.001), showing that symptoms of addiction to UPFs act as a mediator in the relationship between perceived COVID-19-related stress and negative emotional eating. Moreover, the proportion of the mediating effect was 0.64 (*p* = 0.01), meaning that 64% of the influence of perceived stress during the COVID-19 pandemic on emotional eating was mediated by symptoms of addiction to UPFs.

Finally, we examined whether our data aligned with the literature on the relationship between food addiction symptoms and the consumption of UPFs. A positive correlation was found between food addiction symptoms and the reported consumption of UPFs (rho = 0.121; *p* = 0.01), whereas no correlation was found between food addiction symptoms and the consumption of unprocessed foods (rho =  − 0.009; *p* = 0.85). These results suggest that food addiction refers to symptoms of substance-based addiction, specifically to UPFs, as previously postulated (Gearhardt & Schulte, 2021; Monteiro & Cannon, 2023; Schulte et al., 2015).

## Discussion

This study aimed to investigate the mediating role of symptoms of addiction to UPFs in the relationship between perceived stress due to the COVID-19 pandemic and emotional eating. Our findings suggested that symptoms of addiction to UPFs played a significant role as a mediator in the sample of undergraduate Brazilian students: symptoms of food addiction were responsible for 64% of the effect. These results confirm our hypothesis that perceived stress during the COVID-19 pandemic influenced college students’ emotional eating behaviour through food addiction symptoms, which are related specifically to UPF consumption.

Although previous studies have investigated the association between emotional eating and the consumption of palatable food (Dallman et al., 2003; Macht & Mueller, 2007; van Strien et al., 1986), the reasons why emotional eating may lead to the consumption of energy-dense and palatable foods remain unknown. The results of the present study highlight the role of food addiction symptoms in stress-driven emotional eating: the greater the number of symptoms of food addiction is, the higher the negative emotional eating score. Interestingly, negative emotional eating scores were positively associated with food addiction symptoms, whereas positive emotional eating scores were not (Table 2). Researchers have debated whether positive feelings associated with palatable food may lead to positive reinforcement behaviour (Meule & Kübler, 2012; Minhas et al., 2021). Our findings support the notion that food addiction is linked to a coping mechanism for unpleasant feelings instead of a response to positive feelings (Fischer et al., 2004). The daily consumption of palatable food may result in tolerance and dampening of the brain’s reward circuitry and the compulsive seeking of palatable food despite potentially unpleasant consequences (Parylak et al., 2011). In agreement with this hypothesis, individuals with food addiction symptoms do not seem to expect positive reinforcement through eating (Meule & Kübler, 2012). Alternatively, UPF intake may lead to negative reinforcement because of ‘comforting’ effects that might acutely reset organism responses to stress. Our results indicate that negative reinforcement models are important in understanding food addictive eating patterns (Fischer et al., 2004).

Interestingly, this is the first study to examine the psychological stress caused by the COVID-19 pandemic, food addiction symptoms and negative emotional eating together in a mediation model. The COVID-19 pandemic played a major role in triggering mental health problems linked to stress (Cénat et al., 2021; Portugal et al., 2022; Santomauro et al., 2021). Our findings agree with the literature showing that perceived stress is associated with food addiction symptoms (Hong et al., 2020; Luo et al., 2022; Najem et al., 2020; Wei et al., 2019) and emotional eating (Devonport et al., 2019; Macht & Mueller, 2007; Najem et al., 2020; van Strien et al., 2012, 2013, 2014). However, previous studies have investigated these factors separately. Our study provides new evidence that negative reinforcement plays a crucial role in food addiction processes by revealing that food addiction is a mediator of the relationship between stress and emotional eating due to negative emotions (and not due to positive emotions).

The consumption of palatable foods, such as UPFs, has comforting effects that can initially relieve negative feelings and stress (Macht & Mueller, 2007). In fact, during the COVID-19 pandemic, the consumption of UPFs increased (Andrade et al., 2023). However, repeated, intermittent intake of UPFs may instead lead to increased activation of stress-related brain circuits and downregulation of reward pathways in the brain (Parylak et al., 2011). In this way, individuals may need more palatable food to deal with negative feelings, creating a food addiction scenario. Preventing the onset of food addiction symptoms by reducing the consumption of UPFs may be an important strategy to break this cycle.

### Implications for research and practice

To our knowledge, this is the first study to examine the associations between perceived stress during the COVID-19 pandemic, symptoms of addiction to UPFs and emotional eating via a mediation analysis. One of the goals of a mediation analysis is to elucidate possible mechanisms of hypothetical interventions that target the exposure (perceived stress during the COVID-19 pandemic) or mediator (symptoms of addiction to UPFs) promoting changes in the dependent variable (negative emotional eating) (Liu & Ulrich, 2016). Thus, the results can be utilised to support interventions and the adoption of preventive measures aimed at reducing the psychological stress consequences of the COVID-19 pandemic.

To address the emotional eating issue among university students, public health intervention programs should address psychological distress as well as signs of addictive eating behaviour in this population. Since psychological distress increased after the COVID-19 pandemic (Santomauro et al., 2021; WHO, 2022), initiatives that strengthen psychological and psychosocial support for people in distress might also minimise the consequences of stress, such as emotional eating. The development of new or adapted psychological intervention strategies is needed to improve resilience and to treat or prevent the stress-related consequences triggered by the COVID-19 pandemic (WHO, 2022). For example, psychological internet-based interventions seem to be effective in reducing stress among college students (Malinauskas & Malinauskiene, 2022).

Our results revealed that symptoms of food addiction are an intermediate variable (mediator) that is influenced by psychological stress and, in turn, influences negative emotional eating. The symptoms of food addiction were shown to be associated with UPF consumption but not with unprocessed food intake in our study. A study conducted with Brazilian students also revealed that students with food addiction had a greater intake of UPFs and a lower intake of unprocessed or minimally processed food than did students without food addiction (da Silva Júnior et al., 2022). The increased intake of UPFs may result in lower diet quality, which robustly increases the risk of noncommunicable chronic diseases, including type 2 diabetes, cancer and cardiovascular diseases (Lane et al., 2021). As food addiction is linked to the consumption of UPFs, intervention strategies may focus on reducing UPF consumption.

In parallel with strategies for reducing stress, developing healthier food environments, with less availability of UPFs and more availability of unprocessed and minimally processed foods, are important factors to consider in managing food addiction symptoms (Scaciota et al., 2020). One of the sales strategies associated with UPFs is how practical they are, being ready-to-heat or ready-to-eat (PAHO, 2019a). Undergraduate students often do not have the time to prepare their own food, which may increase the consumption of UPFs in this population, especially for students who live outside their family homes (Fondevila-Gascón et al., 2022). Therefore, healthier food environments should be prioritised to encourage healthy habits that can last a lifetime. However, the availability of UPFs is increasingly high and very accessible in universities in Brazil, in contrast to the decreasing supply of unprocessed and minimally processed foods (Franco et al., 2020). In the city of Niterói, where the study was conducted, a law prohibiting the sale, distribution and advertising of UPFs in schools was introduced in 2023 (Niterói, 2023). However, federal universities in the city of Niterói are exempt from municipal legislation. Thus, it is imperative that these initiatives, encompassing all educational settings from municipal to federal, occur at the national level. Since scientific evidence is required to support public policies, our findings help support new regulations that aim to promote food environments with a greater availability of unprocessed and minimally processed foods.

Other public policies and measures to effectively reduce the demand for and the provision of UPFs not only in universities but also in other food environments include the regulation of marketing (PAHO, 2019b) and the implementation of front-of-package labelling. Front-of-package labelling has been recommended as one of the key policy tools for helping reshape the food environment by allowing consumers to clearly and easily identify products containing excessive amounts of nutrients of public health concern, thus helping them make healthier food purchases (PAHO, 2020). In Brazil, new labelling requirements for prepackaged food and beverages include the provision of front-of-package nutrition labelling (Anvisa, 2020), which may contribute to reducing the consumption of UPFs and preventing food addiction symptoms among college students and the overall population in the future.

This study has several limitations. First, the sample was from a public university in Brazil; thus, caution should be exercised in generalising the results to other populations. Second, almost 70% of the participants were female. Even though there is a greater proportion of women than men in public universities in Brazil (INEP, 2022), it would be interesting to have a more diverse (e.g. a larger age range) and balanced population in the future. Furthermore, future research should investigate a more complex and multifactorial model that considers other variables as mediators. We focused the analyses on a nonclinical population to facilitate interpretation of the data in terms of emotional eating and food addiction symptoms. Therefore, our model does not allow us to make direct inferences between perceived stress during the COVID-19 pandemic and eating disorders. However, since emotional eating and food addiction increase the risk for eating disorders (Rossi et al., 2020; Wiss, 2022), preventing the onset of food addiction symptoms via a decrease in UPF intake may have impacts not only on emotional eating but also on the development of eating disorders.

## Conclusion

The study revealed that addiction to UPFs mediates the relationship between COVID-19-related psychological stress and negative emotional eating behaviour. Healthier food environments encompassing all educational settings, from municipal to federal settings, are recommended to reduce emotional eating behaviour among college students in Brazil.

## Supplementary Information

Additional file 1. Supplemental material.

## Data Availability

The datasets generated and/or analysed during the current study are available in the Mendeley Data repository: ‘Dataset: Addiction to ultra-processed foods as a mediator between psychological stress and emotional eating during COVID-19 pandemic’, Mendeley Data, V1, 10.17632/xs9fbf2j6r.1.

## Abbreviations

UPFs: Ultra-processed foods
mYFAS 2.0: Modified Yale Food Addiction Scale 2.0
CSM: Coronavirus Stress Measure
EMAQ: Emotional Eating Questionnaire
DSM-5: Diagnostic and Statistical Manual of Mental Disorders, Fifth Edition
BMI: Body mass index
