# Supplementary material for: Addiction to ultra-processed foods as a mediator between psychological stress and emotional eating during the COVID-19 pandemic
Source: Psicol Reflex Crit. 2024 Sep 18;37:39. doi: 10.1186/s41155-024-00322-1 (PMC11410752; doi:10.1186/s41155-024-00322-1)
Supplement: Supplementary file 1 — Additional file 1. Supplemental material. [file 41155_2024_322_MOESM1_ESM.docx]

**Supplemental Material**

**Confirmatory Factor Analysis**

We carried out a Confirmatory Factor Analysis (CFA) using Lavaan (an R program; Rosseel, 2012). We present the results of two estimators, as the fit indices may be influenced by the estimation method employed (Shi and Maydeu-Olivares, 2020; Xia and Yang, 2019). First, the diagonally weighted least square mean and variance adjusted (WLSMV) estimator, which is recommended for ordinal response scales (Hancock and Mueller, 2013; Muthén, 1993), was employed and items of all scales were treated as categorical. Additionally, we report indices obtained with the robust maximum likelihood (MLR) estimator, and its associated robust fit indexes. The YFAS' CFA model was based on a single factor model (Schulte & Gearhardt, 2017). The stress scale CFA model was likewise based on a one-factor model (Arslan et al., 2021), whereas the appetite scale used a two-factor model (Nolan et al., 2010). Items were specified to a single factor only, and the latent variables were specified to correlate with one another.

The fit indices we employed were the comparative fit index (CFI), Tucker-Lewis Index (TLI), root mean squared error of approximation (RMSEA), and standardized root mean squared residual (SRMR). Acceptable fitting models had a CFI and TLI ≥ 0.90, SRMR ≤ 0.08, and RMSEA ≤ 0.10, whereas close-fit models had values greater than.95 for the TLI and CFI and 0.05 or less for the RMSEA (Hu and Bentler, 1995; Whittaker, 2016).

Results

*Yale Food Addiction Scale 2.0 (YFAS)*

YFAS results indicate that the one-factor model presented reasonably good fit in the current study (WLSMV estimator: CFI= 0.97, TLI= 0.96, RMSEA= 0.08, SRMR= 0.06; MLR estimator: CFI= 0.91, TLI= 0.90, RMSEA= 0.08, and SRMR = 0.05). Factor loadings are presented in Table S1. Factorial loadings are good or acceptable in general except for two items when using the MLR estimator.

Table S1: Confirmatory factor analysis: standardized factor loadings for the YFAS scale.

| YFAS  (one-factor model) | | | |
| --- | --- | --- | --- |
| Factor loadings | | | |
|  | MLR estimator | WLSMV estimator |  |
|  |  |  |  |
| Item 1 | 0.61 | 0.70 |  |
| Item 2 | 0.55 | 0.61 |  |
| Item 3 | 0.31 | 0.58 |  |
| Item 4 | 0.70 | 0.73 |  |
| Item 5 | 0.80 | 0.88 |  |
| Item 6 | 0.72 | 0.82 |  |
| Item 7 | 0.65 | 0.87 |  |
| Item 8 | 0.73 | 0.82 |  |
| Item 9 | 0.69 | 0.74 |  |
| Item 10 | 0.67 | 0.76 |  |
| Item 11 | 0.61 | 0.67 |  |
| Item 12 | 0.19 | 0.47 |  |
| Item 13 | 0.50 | 0.57 |  |
|  |  |  |  |

*Coronavirus Stress Measure (CSM)*

Data from the CSM scale also presented a good fit (WLSMV estimator: CFI= 0.99, TLI= 0.97, RMSEA= 0.08, SRMR= 0.04; MLR estimator: robust CFI= 0.97, TLI= 0.95, RMSEA= 0.10, and SRMR= 0.08) to the one-factor model proposed by Arslan et al. (2021). Factor loadings are presented in Table S2. All factorial loads are above 0.50.

Table S1: Confirmatory factor analysis: standardized factor loadings for the CSM scale.

| CSM  (one-factor model) | | | |
| --- | --- | --- | --- |
| Factor loadings | | | |
|  | MLR estimator | WLSMV estimator |  |
|  |  |  |  |
| Item 1 | 0.62 | 0.70 |  |
| Item 2 | 0.78 | 0.82 |  |
| Item 3 | 0.73 | 0.79 |  |
| Item 4 | 0.77 | 0.83 |  |
| Item 5 | 0.83 | 0.88 |  |
|  |  |  |  |

*Emotional Eating Questionnaire (EMAQ)*

CFA results obtained from the EMAQ scale indicate that the two-factor model presented an acceptable fit in the current study (WLSMV estimator: CFI= 0.96, TLI= 0.96, RMSEA= 0.11, SRMR= 0.09; MLR estimator: CFI= 0.85 TLI= 0.83, RMSEA= 0.09, and SRMR = 0.09). All factorial loads are above 0.50, except for one item (0.49).

Table S3: Confirmatory factor analysis: standardized factor loadings for the EMAQ scale.

| EMAQ  (two-factor model) | | | |
| --- | --- | --- | --- |
| Factor loadings | | | |
|  | MLR estimator | WLSMV estimator |  |
|  |  |  |  |
| Factor1 |  |  |  |
| Item 1 | 0.71 | 0.78 |  |
| Item 2 | 0.49 | 0.50 |  |
| Item 4 | 0.67 | 0.70 |  |
| Item 5 | 0.68 | 0.76 |  |
| Item 7 | 0.74 | 0.76 |  |
| Item 8 | 0.52 | 0.56 |  |
| Item 9 | 0.72 | 0.82 |  |
| Item 10 | 0.51 | 0.57 |  |
| Item 13 | 0.65 | 0.70 |  |
| Item 15 | 0.66 | 0.72 |  |
| Item 16 | 0.74 | 0.83 |  |
| Item 17 | 0.59 | 0.70 |  |
| Item 19 | 0.63 | 0.65 |  |
| Item 21 | 0.66 | 0.72 |  |
|  |  |  |  |
| Factor 2 |  |  |  |
| Item 3 | 0.66 | 0.66 |  |
| Item 6 | 0.79 | 0.80 |  |
| Item 11 | 0.72 | 0.74 |  |
| Item 12 | 0.85 | 0.86 |  |
| Item 14 | 0.88 | 0.89 |  |
| Item 18 | 0.48 | 0.49 |  |
| Item 20 | 0.70 | 0.72 |  |
|  |  |  |  |
| Item 22 | 0.70 | 0.72 |  |
|  |  |  |  |

**Yale Food Addiction Scale items**

1. I ate to the point where I felt physically ill
2. I tried and failed to cut down on or stop eating certain foods
3. I spent more time feeling sluggish or tired from overeating
4. I avoided work, school or social activities because I was afraid I would overeat there
5. I kept eating in the same way even though my eating caused emotional problems
6. Eating the same amount of food did not give me as much enjoyment as it used to
7. If I had emotional problems because I had not eaten certain foods, I would eat those foods to feel better.
8. My friends or family were worried about how much I overate.
9. My overeating got in the way of me taking care of my family or doing household chores.
10. I was so distracted by eating that I could have been hurt (e.g. when driving a car, crossing the street and operating machinery).
11. I had such strong urges to eat certain foods that I could not think of anything else.
12. I had significant problems in my life because of food and eating. These may have been problems with my daily routine, work, school, friends, family or health.
13. My eating behaviour caused me much distress.

**Coronavirus Stress Measure items**

1. How often have you been upset because of the COVID19 pandemic?
2. How often have you felt that you were unable to control the important things in your life due to the COVID19 pandemic?
3. How often have you felt nervous and stressed because of the COVID19 pandemic?
4. How often have you found that you could not cope with all the things that you had to do due to the COVID19 pandemic?
5. How often have you felt difficulties were piling up so high that you could not overcome them due to the COVID19 pandemic?

**Coronavirus Stress Measure items**

Emotional Domain

1. Sad
2. Bored
3. Confident
4. Angry
5. Anxious
6. Happy
7. Frustated
8. Tired
9. Depressed
10. Frightened
11. Relaxed
12. Playful
13. Lonely
14. Enthusiastic

**Scatterplots**


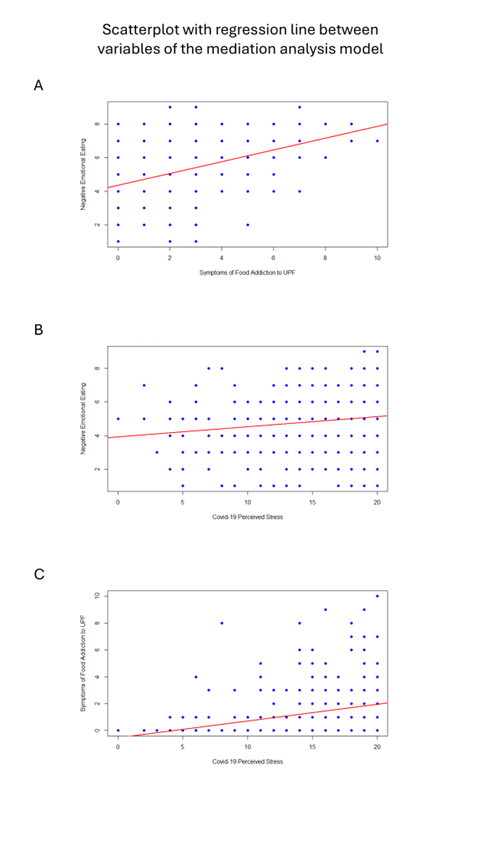


**Hayes’s PROCESS Model 4**

A mediation analysis was conducted using Hayes's PROCESS model 4, with BMI controlled for in the model. The results showed consistency with only minor variations in the coefficients. The 'a' path (B = 0.127, *p* < 0.001) and 'b' path (B = 0.297, *p* < 0.001) remained significant, while the direct effect (B = 0.018, *p* = 0.36) was not significant. Both the total effect (B = 0.051, *p* = 0.01) and the indirect effect (B = 0.04, CI [0.02, 0.05]) were significant, reinforcing the presence of a mediation effect.

**References**

Hancock, G. R., & Mueller, R. O. (2013). Structural equation modeling: A second course, 2nd ed (p. xxvii, 673). IAP Information Age Publishing.

Hu, L., & Bentler, P. M. (1999). Cutoff criteria for fit indexes in covariance structure analysis: Conventional criteria versus new alternatives. Structural Equation Modeling, 6(1), 1–55. <https://doi.org/10.1080/10705519909540118>

Muthén, B. (1993). Goodness of Fit with Categorical and Other Non-Normal Variables. In K. A. Bollen, & J. S. Long (Eds.), Testing Structural Equation Models (pp. 205-243). Newbury Park, CA: Sage Publications.

Rosseel, Y. (2012). lavaan: An R Package for Structural Equation Modeling. Journal of Statistical Software, 48, 1–36. <https://doi.org/10.18637/jss.v048.i02>

Rutkowski, L., & Svetina, D. (2014). Assessing the Hypothesis of Measurement Invariance in the Context of Large-Scale International Surveys. Educational and Psychological Measurement, 74(1), 31–57.<https://doi.org/10.1177/0013164413498257>

Shi, D., & Maydeu-Olivares, A. (2020). The effect of estimation methods on SEM fit indices. Educational and Psychological Measurement, 80(3), 421–445. <https://doi.org/10.1177/0013164419885164>

Whittaker, T. A. (2016). ‘Structural equation modeling’. Applied Multivariate Statistics for the Social Sciences (6th ed.). Routledge: New York. 639-746.

Xia, Y., Yang, Y. RMSEA, CFI, and TLI in structural equation modeling with ordered categorical data: The story they tell depends on the estimation methods. Behav Res 51, 409–428 (2019). <https://doi.org/10.3758/s13428-018-1055-2>
